# The Medical Aftermath of the War

**Published:** 1919-01

**Authors:** 


### WAR MEDICINE

Published by the American Red Cross Society in France
for the
Medical Officers of the American Expeditionary Forces

**EDITORIAL OFFICES :** **2, Place de Rivoli, Paris, Rooms 422-425**

WAR MEDICINE *accepts no original articles. Its scope is limited to the publication of the proceedings of the Research Society of the American Red Cross in France, abstracts of original articles, and editorial comment on subjects pertaining to the medicine, surgery, and hygiene of the war.*

*Circulars, bulletins, and reports from the Office of the Chief Surgeon of the American Expeditionary Forces will also appear in* WAR MEDICINE.

WAR MEDICINE *is distributed free of charge to the medical officers of the American Expeditionary Forces.*

*All communications pertaining to the mailing list should be addressed to the Editorial Offices. If copies are not received promptly and regularly the managing editor should be informed at once. Addresses should be written distinctly.*

THE RESEARCH SOCIETY OF THE AMERICAN RED CROSS IN FRANCE
*All communications should be addressed to the Secretary,*
*2, Place de Rivoli, Paris, Room 422*

### EDITORIAL COMMENTS

### THE MEDICAL AFTERMATH OF THE WAR

An infantry lieutenant tells of overhearing a conversation between two privates, in which one said to the other : "What are you going to do after the War?" The reply was : "I am going back to Indiana and spend the rest of my life outlying the G. A. R.'s. Apropos of this is the story of the medical officer who wrote home that he was so accustomed to the hospital in which he was working being bombed by the Germans, that, without any thought of danger, he "operated upon wounded soldiers by the light of the bursting shells". His people were so proud of the "hero" of the family, that the letter was given to the local editor for publication. The world being small, a copy of the weekly paper from this small town somewhere in the United States was received by another member of the hospital staff in France, and the young medical officer is still explaining that he "never said it".

A celebrated American physician who could not enter the Medical Reserve Corps because of a physical disability said that one of the regrets which he had in having to stay at home was that "after the war the fellows who went to France will do all the talking, and those of us who did not go will have to listen to them for the rest of our lives". He evidently had vision, because those who came to France have seen so many remarkable things that it will be but natural for them to entertain their friends at home with the recitation of facts, which, without added imaginary touches, make wonderfully interesting stories.

The physicians who have had army experience in France will not talk all the time, however, when they get home, because they will soon be busier practising medicine and surgery than they were before they entered the Medical Reserve Corps. They will be poorer in purse, but they will go back rich in experiences that will be of the utmost help to them in their daily work. They will have the confidence and respect of the people of their communities and will therefore have greater prestige, because, by giving up established practices to serve their country, they have shown that they hold duty higher than dollars.

### A Broader and Better Medical Profession.

There is no question that the American people appreciate the unselfish spirit that prompted the best men in the medical profession to give up the comforts of home, the joy of family and friends, and the emoluments of an established clientele, to serve their country in the greatest crisis in history; and the public has been informed through the newspapers and magazines of the advances that have been made in all the branches of medicine and surgery as a result of the opportunity to study the various phases of wounds and diseases on a larger scale than ever before. The medical profession will therefore come out of the war as the most respected of the learned vocations. The spirit of the time is that "he profits most who serves best". The service of the medical men in the war has been wholly unselfish, and they will be rewarded by enjoying the confidence and patronage of the fathers, mothers, relatives and friends of the soldiers whose health and lives they safeguarded.

Aside from the prestige and remuneration that will come to the doctors who served as military officers, they will be better men and better doctors through having been associated with others in their profession as they have in army life. It happens only too often that medical men in villages and large cities, if they are fortunate, develop an "overweening" conceit because of their success, while the less favored sometimes come to think that the physician or surgeon who builds up a good practice does so by employing unprofessional tactics. Then there is the jealousy of the successful ones among each other, and in the cliques that surround them in every city. There will be less of envy and jealousy and a more tolerant spirit among physicians and surgeons after the war than ever before, because they have been associated with so many doctors whom they know have worked unselfishly, and they have had the intimate contact with their professional brothers that broadens thought, and makes men act with greater kindness and generosity towards one another.

### Community Hospital.

Medical men, particularly in small towns, have suffered from the lack of co-operation. There may be in a town four, five, or eight or ten good doctors, one or two of whom have had good surgical training; another may be a good diagnostician, while another is better fitted for laboratory work; but because they have not pulled together, none of them have done the thorough and scientific work of which they are capable. As a result, when their good-paying patrons needed expert medical and surgical attendance, they have been sent to the cities for diagnosis and treatment. The medical men who have served in the Army have seen as good surgery in tents, shacks, or in residences converted into temporary hospitals, as they have in the finest surgical amphitheatre in the great medical centers. They have not only seen as good work done, but many who never had the opportunity of doing surgery before have been trained in surgical technic by association with the masters in surgery who have been in France, and have learned that they can perform the major operations as well as their teachers.

When these men go home, they will form into clinical groups, they will build up and equip inexpensive hospitals, they will take post-graduate courses in the line of work in which they are best prepared to specialize, and they will be prepared to give their patrons as good medical and surgical attention as they could get in the larger cities. They will be more thorough and accurate in their work. For instance, the diagnosis of appendicitis will be made earlier and operated upon in the village hospital without submitting the acute appendix cases to the dangers of delay and travel that have cost many a life.

### Team Work.

The constant association with each other of internists, surgeons, roentgenologists, bacteriologists, pathologists and medical men in other lines of work, in the thorough work that has been done, particularly in the base hospitals, will make men engaged in practicing all branches of medicine desire to give their patients at home the same attention. Consequently, they will arrange to be associated with similar groups of specialists when they begin private work again. They will realize that general practitioners in the future, more than ever before, will refer their cases to men who can give them the benefit of such group consultations.

The general practitioner, the keystone of the arch upon which the whole foundation of medicine rests, will come out of the Great War bigger, broader and more resourceful than ever before. In the past he has been overworked, and only too often did not have the time to study his cases thoroughly, and for that reason has not been as accurate in his diagnoses as he should have been. In many instances, he has not availed himself of the advantages of the laboratory. In army work he has had the opportunity as never before of seeing cases studied with the aid of the laboratory and the X-ray, and with the co-operation of dentists, ophthalmologists, neurologists, urologists and other specialists. When he gets back home, he will see to it that his patients get the benefit of the aids in diagnosis and treatment to which he has become accustomed in army work.

Laboratory and X-ray men have also profited by their association with general practitioners, internists and surgeons, and they will not be so "cocksure" of their diagnoses without clinical assistance. In other words, medical men have learned the advantages of and necessity for "team" work, that will be carried into their private work, and their patients will profit thereby, while they themselves will also be gainers by co-operating with their medical confreres in all branches of medicine and surgery.

### The Surgeons.

The great surgeons of the United States, and the best men in all branches of medicine, with few exceptions — and most of those who did not ask for commissions were over-age or had physical disabilities — volunteered their services for army work; and they have given the best that was in them in treating our sick and wounded soldiers. Indeed, there has been no finer spirit of patriotism manifested than that of the surgeons of the United States, who in many instances closed up their private hospitals and gave up incomes amounting to many times the salary of a major, the highest commission in the Army to which a Medical Reserve Corps officer could attain in the first year of the war, and asked for nothing except the opportunity to give their services to the men who were to fight their country's battles. And the surgeons have served faithfully and well; but, while serving, they too have profited by their army work. The contact with others has broadened their viewpoints, and they have had experiences, particularly in bone and emergency surgery, which will be helpful to them in their work at home.

A prominent surgeon said that one of the great things that has accrued to surgery from the war has been the more general use of nitrous oxide and oxygen anesthesia, which, if given properly (three-fourths nitrous oxide to one fourth oxygen), actually raises blood-pressure and prevents or lessens shock, thereby decreasing the danger of surgical anesthesia. He also said that the advent of the trained female anesthetist is one of the permanent benefits to surgery that has come from the war.

The leaders in surgery have discovered many first-class surgeons of whom they had never heard before; and they have been frank enough to admit that the best surgeons are not all working in the great surgical centers, but that there are many good operators in all parts of the country. There will always be men who, because of great achievement, will be recognized as leaders of their profession, but the war has been a great leveler of surgeons as well as of men in all lines of work. It has developed the latent talents of many well-trained men, who after the war will take their places as among the most competent surgeons of the century.

It is no doubt true that the Army will turn out some "half-baked" surgeons who will develop the "furor operativus" and be a menace to their communities; but it will not take their patrons long to learn that with the surgical tyro "the paths of glory lead but to the grave" and amateur surgery will prove unprofitable and fall into deserved disrepute.

### The Impetus to Research Work.

American laboratory workers were not in the war long enough to accomplish a great deal in medical research, though the original studies and investigations, notably on trench fever, have made important contributions to the science of medicine and surgery. A number of surgical problems were being investigated in France on a large scale when the war closed. There can be no question, however, that the war has stimulated research work on many lines and that it will be continued in the laboratories of the United States as well as in those of our Allies. The public has received some needed education in the importance of research work on many lines, and it will be easier to induce legislators and philanthropists to provide funds to establish new research laboratories and to develop those that already are doing such good work. The contact with the research men of Great Britain and France has stimulated our men to greater efforts; and we have learned from them that it is the duty of Government to provide greater facilities for laboratory and research work, and appropriations for such purposes will be easier to obtain in the future.

### Venereal Diseases.

The war has brought to the attention of the public the fact that venereal diseases are among the greatest public health problems. Actual demonstrations have been made, which have proved to the satisfaction of all unprejudiced men that houses of prostitution are centers of infection that cannot be tolerated, and public officials in every city in the United States will be forced to regard organized vice as a health problem. As a result of the war the United States Government has appropriated $1,000,000 to be allotted proportionately to the various states, provided that they add an amount equal to that given them for work in preventing venereal diseases. When American physicians get back home, they will find genito-urinary dispensaries and prophylactic stations everywhere.

The public will understand, better than ever before, the necessity for the accurate diagnosis, and the thorough and scientific treatment of gonorrhoea and syphilis, with their complications and sequellæ; and though there will be a decrease in venereal diseases, the urologists will be busier than ever before. The specialty of urology will, therefore, attract many men now in general practice, and it will become of greater importance than ever before.

### Preventive Medicine.

There have not been many important advances made in the methods of preventing disease as a result of the war, but an impetus has been given to public health work that will last long after the soldiers have returned to their homes. More than 3,000,000 citizens of the United States have been taught something about military hygiene, which differs but little from that of civil life. They will go back to their homes where they have tolerated many sanitary crimes because they did not know better. They will not only live more hygienic lives themselves, but they will see to it that their neighbors do not harbor infection on their premises. Doctors from civil life have learned much about preventive medicine, which on their return to civil practice they will employ in private work. Then the Army has trained a large number of sanitarians who will be available for public health work after the war.

The epidemics that have occurred incident to bringing together large bodies of men, and the fact that approximately twenty-five percent of the young men of our country are not fitted for military service because of physical disabilities, have taught the public the importance of preventive medicine. The draft law also brought out the necessity for accurate birth statistics, and among the aftermaths of the war may be the assurance that we shall have more reliable vital statistics. In other words, the war has helped tremendously to get an enlightened public opinion on health matters that is essential to success in carrying out methods for preventing communicable diseases, and as a result Congress will not delay much longer the needed legislation providing for a National Department of Health with a sanitarian in the President's Cabinet.

All of the advances in the science and practice of surgery resulting from the war cannot be mentioned in an editiorial comment. There is no question, however, that mankind has been and will be the gainer by the efforts of American physicians who have sacrificed to serve.

